# Virtual postgraduate orthopaedic practical examination: a pilot model

**DOI:** 10.1136/postgradmedj-2020-138726

**Published:** 2020-10-03

**Authors:** Karthikeyan P Iyengar, Vijay Kumar Jain, Raju Vaishya

**Affiliations:** Department of Orthopaedics, Southport and Ormskirk NHS Trust, Southport, UK; Department of Orthopaedics, Atal Bihari Vajpaee Institute of Medical Sciences, New Delhi, India; Orthopaedics, Indraprastha Apollo Hospital, New Delhi, India

**Keywords:** Medical education & training

## Abstract

COVID-19 pandemic has had a profound impact on the delivery of medical education, training and examination schedule across the world both at undergraduate and at postgraduate (PG) levels. The novel coronavirus SARS-CoV-2 outbreak has resulted in the cancellation of traditional in-person meetings and clinical examination assessments, learning and education activities because of concern of viral transmission. Various medical universities, Royal Medical and Surgical Colleges in the UK have suspended delivery of qualifying examinations until they can be resumed safely with updated social distancing guidelines. This article evaluates the role and the possibility of virtual PG practical examination template based on authors' own recent experience of conducting successful virtual practical PG orthopaedic qualifying examinations during the COVID-19 pandemic in New Delhi, India. Advances in telecommunication technology can enable academic institution and orthopaedic educators to develop such a model and act as a blueprint for the future.

## Introduction

The highly contagious novel coronavirus SARS-CoV-2 outbreak has necessitated a reduction of ‘face-to-face’ interactions and various infection prevention strategies including lockdown, social distancing measures and appropriate use of personal protective equipment to prevent viral transmission.^1 2^

Healthcare systems across the world have adapted, reorganised and restructured the delivery of healthcare to patients during the COVID-19 pandemic.^3^ Routine elective orthopaedic services have been suspended with ‘face-to-face’ clinic appointments being replaced by telemedicine virtual consultations to support the public health crisis.^4^ Teaching hospitals and regional deaneries have rapidly moved towards remote learning using online platforms and webinars. These actions have been found to be at least as effective as other methods of training.^5^ Qualifying exit undergraduate (UG) and postgraduate (PG) examinations are necessary elements of a medical doctor’s career progression. In the UK, COVID-19 has prevented the preparation and organisation of traditional ‘face-to-face’ practical clinical and viva voce examinations at both the UG and PG levels. Proposed examination scheduled to be held in the spring of 2020 has been suspended until the near future following guidelines issued by the different Royal Medical and Surgical institutions.^6^ The trainees have been regularly updated about developments, and plans are afoot to resume examination activity from September 2020.^7^

This resumption in the examination activity is being planned with the expectation that social distancing measures will remain in place and will allow the safety of candidates, examiners and staff members. Hence, there is uncertainty about how practical examinations are going to unfold in the near future.

The use of Video-Projected Structured Clinical Examination instead of the traditional oral (viva voce) examination in the assessment of final year medical students has been found to be an effective replacement in the past.^8^

Advances in telecommunication technology and computer software programs can provide us with an opportunity to apply these to conduct a virtual practical clinical assessment model for the PG candidates appearing for subspeciality examinations during the current pandemic and social distancing restriction guidelines, which we are likely to continue in the future.

We propose a virtual PG practical orthopaedic examination template as a tentative blueprint for such examinations in the UK building on the authors own experience, in India. We believe a validated model can then be applied to other subspecialities.

## Traditional Orthopaedic Postgraduate Frcs (TR and ORTH) Exam

The PG qualifying examination for Trauma and Orthopaedics in the UK is the FRCS (Tr and Orth). It is overseen by the Joint Committee on Intercollegiate Examinations (JCIE). Criteria and guidance apply for the exam via the JCIE website (www.jcie.org.uk). The FRCS (Tr and Orth) examination has a set format. It consists of two parts: Part 1 is the written section, delivered through computer-based testing system on an electronic format and consists of extended matching item questions and single best answers questions. The candidate qualifies for the second part following a successful result in Part 1.

Part 2 is divided into two sections: (i) clinical examination and (ii) oral table viva voce section. This section requires ‘face-to-face’ interaction between the candidate, patient or simulated patients, and the examiner during the clinical examination section.

The clinical examination section is broadly divided into (i) intermediate clinical examination of two cases: 15 min each, one on upper limb and one on lower limb pathologies. Candidates are expected to present a concise clinical history of the examined case, perform an observed clinical examination, provide a discussion on the approach and management of that particular patient; (ii) six short clinical cases usually divided with three upper limbs and three lower limb pathologies being analysed in 5 min each with focused clinical assessment.

The oral table viva voce section has four examination stations on (i) trauma × 30 min, (ii) adult and pathology × 30 min, (iii) paediatric and hands × 30 min and (iv) basic science × 30 min.

## Current Challenges

COVID-19 has created a situation in which conducting a traditional ‘face-to-face’ part 2 FRCS (Tr and Orth) examination is challenging, impractical and potentially unsafe with the need for infection control and social distancing guidelines. The safety of all those involved in the exam including the candidates, examiners, staff and the patients or examination volunteers acting as patients is of paramount importance. Hence, organisation of examination centres in a socially distanced context to prevent vector transmission is a prerequisite before ‘face-to-face’ evaluation can resume. A ‘zero-patient contact virtual practical exit examination’ for orthopaedic trainees can be explored with the assistance of Information and Communications Technology applications and computer-based platforms based on previous such endeavours.^8 9^

## Strategies and Considerations for the Proposed Pathway

1. Prevention of COVID-19 infection: The first and foremost objective is to prevent COVID-19 infection to the examiners, trainees, staff, patients and people involved with examinations. Maintenance of social distancing guidelines including using appropriate personal protective gears (eg, face mask, disposable gloves, etc) will be mandatory.
2. Collection of clinical images: One of the drawbacks of virtual examination is the lack of multidimensional assessment of the patient. To replicate a real-life clinical patient scenario, the stations will have to be replaced with representative clinical images in a multidimensional and preferably a digital format (eg, in a virtual hip case examination scenario, pictures from the anterior, lateral and posterior aspects, in sitting and lying down position of the patient, can provide a complete inspection finding).^10^ However, it is essential that the patient images are taken, after an informed consent with an explicit explanation given to the patient as to the purpose it will be used for. The undertaking of clinical patient photographs and video recordings must follow the respective National Health Service (NHS) Trust and General Medical Council guidelines to protect patient confidentiality and privacy.^11^
3. Information governance and data protection: While making a digital pool of orthopaedic examination cases, data must be made, stored, transferred, protected or disposed as per data protection laws and NHS digital information governance guidelines to avoid any potential breaches.
4. Collection of videos demonstrating patient’s gait, clinical sign: Collection of videos demonstrating abnormal gait pattern should be part of clinical assessment in the practical section of the examination. A wide range of clinical knowledge can be assessed by showing such videos to the candidates.^12^
5. Preparation of a representative clinical case scenario: A concise clinical history with relevant questions can be put together to make a representative clinical case scenario of disease in such a way that can give clues to reach the proper diagnosis in an examination setting. One example of such a case scenario could be as follows: Tuberculosis of the hip joint in a 12-year-old man, of poor socioeconomic status with a history of low birth weight, presenting with a painful limp with right knee pain on and off for the last 5 months. There could be constitutional symptoms such as weight loss and poor appetite. Examination would reveal wasting of the thigh, limb length discrepancy with decreased abduction, and internal rotation at the hip. The viva can be further built up by asking questions about additional history and examination findings.
6. Use of interactive screen: Use of interactive computer touch screen during the virtual examination can help the examiner to ask the students to draw clinically relevant anatomical angles or to demonstrate pertinent clinical findings (eg, surface making for the site of tenderness in the shoulder and hip pathologies, three-point bony landmarks in the elbow, an illustration of Bryant’s triangle for supra-trochanteric shortening of the femur, demonstration of anterior superior iliac spine and measurement of limb lengthening and wasting, etc). The biomechanical axis of the lower limb can easily be drawn on a computer screen for viva purposes.
7. Copyright requirements: If images being used in the virtual practical examinations are transferred from the internet or websites, the appropriate copyright and the licence agreement will have to be confirmed with the original author.
8. Look, feel and move parts of clinical examination: These three steps form a key element of any clinical examination. Look (inspection) can be easily inferred from clinical photographs and video recordings. However, evaluation of palpation (feel), and range of movement (move) sections of the traditional examination is more difficult to replicate in a virtual practical orthopaedic examination but can be highlighted by innovative, interactive case videos and imaging technology.
9. The anxiety of candidates will be expected as it is a new kind of experience to them. Prior information, orientation and demonstrating representative case scenarios on the examination website portal may help candidates and the examiners to acclimatise to the new concept and formally prepare for the virtual practical examination. The examiners should be reasonably considerate with the examinees in such a virtual exam, being a newer method of assessment.
10. Training: Training and learning of skills in acclimatisation with this new concept of a virtual practical examination will be required. Familiarity with audiovisual technology will allow a smooth experience of the examination process which in itself can be an emotive experience. Supervision with regular review of practice will enhance understanding of the virtual clinical examination model, improve both candidate and examiner satisfaction with the process, and can be expanded to other subspeciality examinations.

**Figure 1:**
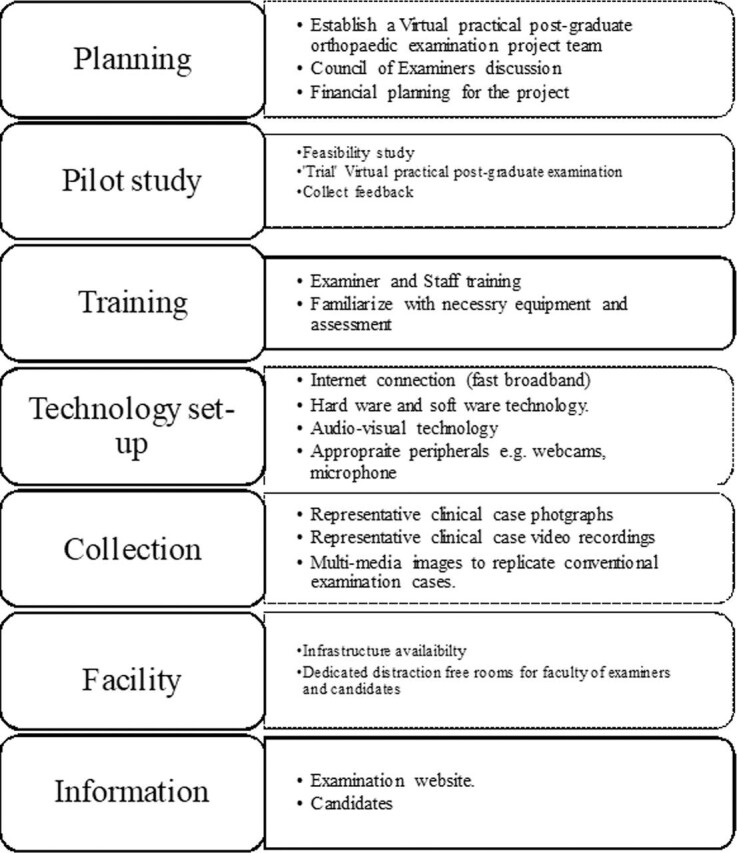
Considerations for setting up a virtual practical postgraduate orthopaedic examination.

**Figure 2:**
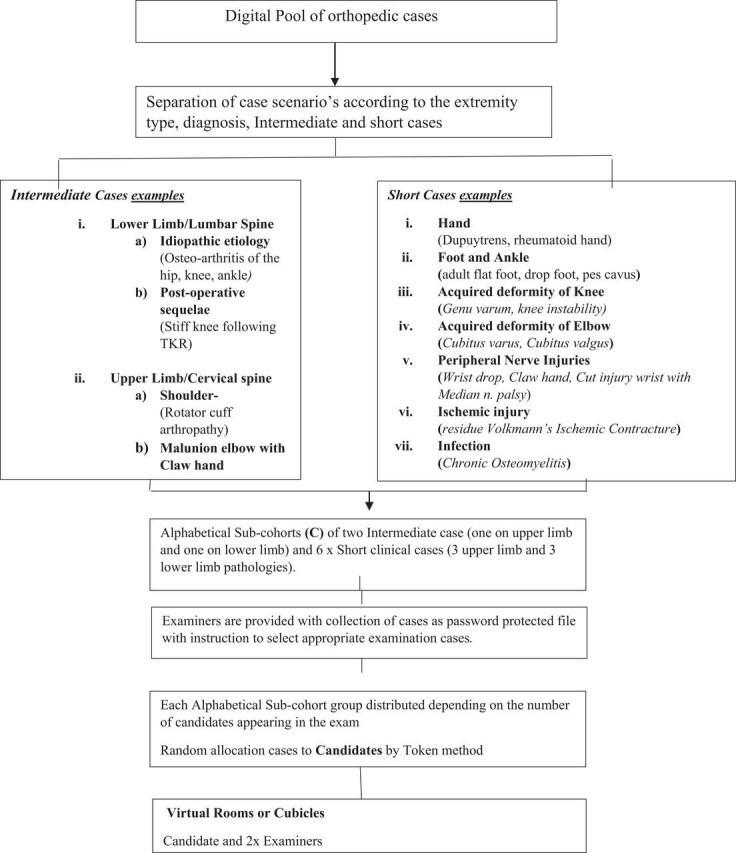
Flow chart showing organisation for virtual postgraduate orthopaedic clinical examination cases based on current FRCS (Tr and Orth) intercollegiate board, UK examination format.

**Figure 3:**
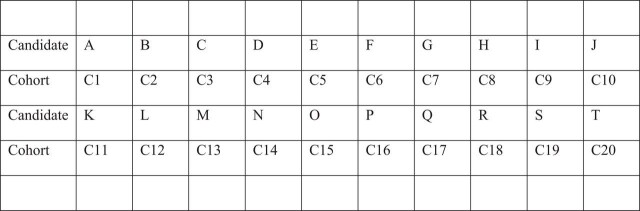
Alphabetical sub-cohorts of Clinical cases.

## Setting up the Virtual Practical Examination and Organisation

1. A virtual digital library of orthopaedic cases with clinical details of patients including a detailed history, examination findings, multidimensional clinical photographs and/or videos with radiology workup is created.
2. Infrastructure—virtual examination cubicles/rooms set up with smart TV and computer and peripherals for digital access.
3. Examiner briefing session.
4. Candidates briefing session including instructions about housekeeping and social distancing guidelines.
5. Intermediate case scenarios.
  - Candidates are provided with information sheets containing brief clinical history and examination findings.
  - Cross-examination of relevant questions to be asked during history undertaken.
  - Pertinent clinical findings leading to diagnosis evaluated.
  - Radiology and role of complementary imaging in arriving at a definitive diagnosis discussed.
6. Short case—the clinical picture of the patient with distinct findings can be provided and candidates asked to make a diagnosis and discuss management.
7. Supplementary resources—radiology imaging (such as X-rays, CT scan, MRI), orthosis prosthesis, instruments, pathological specimens, surgical approaches and osteology can be collected, and presentations can be made on Microsoft PowerPoint. These power points can be distributed to examiners.

## Organisation on The Day

1. Digital pool of clinical cases divided into intermediate and short cases cohorts.
2. Alphabetical subcohorts (eg, A to T) each with 2 intermediate and 6 × short cases.
3. These subcohorts can be distributed to candidates with a token selection system in the morning and afternoon sessions depending on the number of examiners available, number of candidates who are appearing and maintenance of social distancing principles.
4. The candidates can be assessed virtually on the digital collection of clinical cases.

From a recent experience of conducting a virtual orthopaedic PG exam from the premier apex institution of All India Institute of Medical Sciences, New Delhi, India, there was an excellent overall satisfaction rate among the examinees (4.1 out of 5.0) and the examiners (4.5 out of 5.0). Furthermore, the higher scores were reported for questions related to safety of the exam, relevance and quality of the virtual cases, and so on. Hence, it was concluded that the orthopaedic PG exams can be successfully conducted during the COVID pandemic, by virtual means.^13^

## Limitations of Virtual Practical Postgraduate Orthopaedic Examination

There will be a need to ensure quality assurance and improvement of this model. It may be considered an interim solution. Regular audits will need to be undertaken to ensure quality assurance and monitor continual improvement. Serial reviews of practice and ‘surveys’ of experience of the virtual examination model from both the candidates and the examiners will give an insight into the shortcomings and how the virtual exam technique can be improved. Paucity of three--dimensional (3D) evaluation may be another limitation that can be encountered with the virtual concept. However, with the availability of 3D monitors, multidimension imaging and even experience from various ‘gaming’ consoles can be explored to develop interactive virtual reality models to replicate ‘real-life’ patient experience. Doctor–patient relationship is an important part of any exam observed by the examiner and this cannot be assessed on virtual practical exams. Lack of overall assessment (general examination, built, mental status, etc) of a patient is not feasible in a virtual examination setting. However, virtual practical examination model is a novel concept put forward by the authors. The model will surely be developed further to become fair, consistent form of assessment as the process is refined.

## Conclusion

It is acknowledged that the SARS-CoV-2 outbreak had a profound effect on the UG and PG training and education across the world. As COVID-19 pandemic currently restricts the possibility of traditional ‘face-to-face’ practical examination schedules due to social distancing guidelines, innovative models using information and communications technology applications and computer-based programmes as illustrated in this article can be a blueprint for virtual practical examination techniques in the future. It may not replace the traditional ‘face-to-face’ practical examination assessment, but as confidence in the virtual examination model improves, it can be a useful adjunct to the former.

**Main messages**

- Social distancing and infection prevention guidelines due to COVID-19 have necessitated the need to discover alternatives to conventional ‘face-to-face’ practical postgraduate orthopaedic examination techniques.
- Virtual practical postgraduate orthopaedic examination model can be investigated to facilitate candidate evaluation.
- Advances in telecommunication technology including 3D monitors, multidimension imaging can be explored to develop interactive virtual reality models to replicate ‘real-life’ patient experience during the virtual practical examination model.
- Comparative studies between traditional and virtual modes of conducting practical postgraduate examination models will have to be undertaken to ensure an even platform of assessment.

**Current research questions**

- How to develop validated tools to assess such a virtual practical examination model?
- How do we develop a standardisation and consistent evaluation system for the assessment of candidates?
- How to assess impact of reduced face-to-face and hands-on practical examinations?
